# Significant influence of four highly conserved amino-acids in lipochaperon-active sHsps on the structure and functions of the Lo18 protein

**DOI:** 10.1038/s41598-023-46306-6

**Published:** 2023-11-03

**Authors:** Tiffany Bellanger, David da Silva Barreira, Frank Wien, Patrice Delarue, Patrick Senet, Aurélie Rieu, Fabrice Neiers, Paloma Fernández Varela, Sophie Combet, Stéphanie Weidmann

**Affiliations:** 1 Procédés Alimentaires et Microbiologiques (PAM), AgroSup Dijon, PAM UMR A 02.102, Laboratoire VAlMiS-IUVV, Dijon, France; 2 Synchrotron SOLEIL, L’Orme des Merisiers, Saint Aubin BP 48, 91192 Gif-sur-Yvette, France; 3 Laboratoire Interdisciplinaire Carnot de Bourgogne, UMR 6303, CNRS, Université de Bourgogne Franche-Comté, 21078 Dijon, France; 4 Laboratory: Flavour Perception: Molecular Mechanisms (Flavours), INRAE, CNRS, Institut Agro, Université de Bourgogne Franche-Comté, 21000 Dijon, France; 5 CEA, CNRS, Institute for Integrative Biology of the Cell (I2BC), Université Paris-Saclay, 91198 Gif-sur-Yvette, France; 6 Laboratoire Léon-Brillouin (LLB), UMR12 CEA, CNRS, Université Paris-Saclay, 91191 Gif-sur-Yvette CEDEX, France

**Keywords:** Chaperones, Bacteria, Biochemistry, Protein folding, Chaperones

## Abstract

To cope with environmental stresses, bacteria have developed different strategies, including the production of small heat shock proteins (sHSP). All sHSPs are described for their role as molecular chaperones. Some of them, like the Lo18 protein synthesized by *Oenococcus oeni*, also have the particularity of acting as a lipochaperon to maintain membrane fluidity in its optimal state following cellular stresses. Lipochaperon activity is poorly characterized and very little information is available on the domains or amino-acids key to this activity. The aim in this paper is to investigate the importance at the protein structure and function level of four highly conserved residues in sHSP exhibiting lipochaperon activity. Thus, by combining in silico, in vitro and in vivo approaches the importance of three amino-acids present in the core of the protein was shown to maintain both the structure of Lo18 and its functions.

## Introduction

One consequence of environmental stresses is a change in cell membrane fluidity to offset physical stress effects on the membrane^1^. Unfortunately, these stresses inducing modifications can still damage the cell, leading to multiple consequences at the membrane and cytoplasmic level, thus having a very strong impact on the fitness of the bacteria. Lactic acid bacteria (LAB) are not exempt from these consequences, especially since they evolve in harsh environments^2^. For example, the lactic acid bacterium *Oenococcus oeni*, involved in malolactic fermentation, is subjected to various stresses affecting its membrane fluidity, such as acid, alcohol, or thermal modifications^3^. During its evolution, *O. oeni* has developed different strategies to adapt to these stresses, such as the regulation of its internal pH, the modification of its membrane composition and the synthesis of stress proteins^4–7^.

Among the different stress proteins involved in counteracting the deleterious effect of stress, the present study focuses on Lo18, the unique small heat shock protein (sHSP) encoded by *O. oeni*. sHSPs are small proteins ranging from 12 to 35 kDa^8^, with highly heterogeneous primary sequences, especially in the variable terminal domains, but they also contain a conserved α-crystallin domain^9,10^. In the latter domain, a sandwich structure like that found in immunoglobulins, that we called core of the protein, is necessary for their activities^11,12^. Indeed, all sHSPs have been reported to protect against stressed-protein aggregation through their molecular chaperone activity^10,13,14^. In addition, some of them have been found to also interact with the membrane to perform a lipochaperon activity, by maintaining optimal membrane fluidity, crucial for cell survival. For example, HSPA from *Synechococcus sp*.^14,15^, HSP17 from *Synechocystis sp*.^16,17^, HSP17.8 from *Arabidopsis thaliana*^18^ HSP15.8 and HSP16 from *Schizosaccharomyces pombe*^19^ and HSP18.55 from *Lactiplantibacillus plantarum*^20^ have been described as possessing this second activity.

Lo18 is also one of the sHSPs that carries out both these activities. Previous studies have shown that after heat or ethanol stresses, Lo18 interacts as a dimer at the membrane level to rapidly stop increasing membrane fluidity^21–24^. Currently, few data are available in the literature on the molecular mechanisms involved in lipochaperon activity, either for Lo18 or for the other lipochaperon sHSPs^25^.

Recently, it has been observed that all sHSPs with lipochaperon activity have certain highly conserved amino-acids^25^. In order to assess the importance of these amino-acids, four of them (three located in the core of the protein and one located in an adjacent loop) were studied for their possible roles in maintaining the structure and functions of *O. oeni* sHSP Lo18. Proteins modified for these amino-acids were produced. In silico, in vitro and in vivo approaches were then used to characterize these different proteins, notably for their structure and their lipochaperon and molecular chaperon functions. The data obtained indicated that: (i) in the core of the protein, the mutations resulted in a modification of the ratio between α-helices and β-sheets and a decrease of lipochaperon activity; (ii) on the loop between β5 and β7, the mutation affected the protein's structure, insertion and rigidification capacities, without major loss of functions.

## Results

### Key amino-acids for Lo18 lipochaperon activity

**Figure 1:**
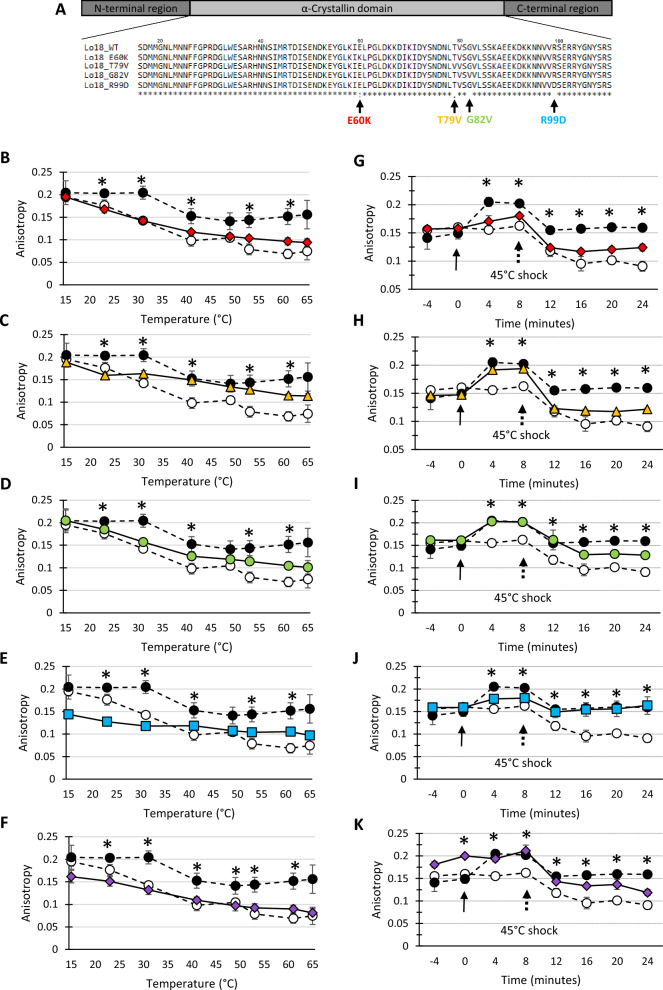
(**A**) Sequence alignment of Lo18 or modified proteins. The alignments are performed on the α-crystallin domain of *O. oeni* Lo18 (accession number CAA67831) or modified after point substitution. The following E60K, T79V, G82V and R99D modifications are indicated with arrows below the sequences. The alignment was performed with MUSCLE (https://www.ebi.ac.uk/Tools/msa/muscle/). Stars, double points and points indicate amino acid residues that are identical in 100%, 80% and 60%, respectively, of all the proteins. (**B**–**K**) Measurement of the fluorescence anisotropy of DPH inserted into O. oeni liposomes during a temperature rise between 16 and 64 °C (**B**–**F**) or after a thermal shock at 45 °C (**G**–**K**). Each panel illustrates liposome fluidisation in the absence of Lo18 (represented by a dotted line and white circles), in the presence of Lo18 WT (represented by a dotted line and black circles), or modified variants of Lo18, namely E60K (red circles) in panels B and G, T79V (yellow diamonds) in panels C and H, G82V (green triangles) in panels D and I, and R99D (blue squares) in panels E and J. Lysozyme (represented by purple diamonds) in panels F and K was used as a negative control in this experiment. For heat shock at 45 °C (**G**–**K**), three phases were defined: the equilibration phase (before 0 min), in which the reaction mixture consists of liposomes and the DPH probe in buffer, the addition of sHSPs (represented by a solid arrow) and the application of heat shock at 45 °C (dotted arrow). The data represent the means and SEs of three independent experiments.

Four proteins with amino-acid substitutions were produced (Fig. 1A) to investigate the protein domain of sHSP involved in membrane interaction. These amino-acids are highly conserved residues among sHSP with lipochaperon activity^25^. The E60K, T79V, G82V and R99D substitutions of Lo18 correspond to the main conserved residues of the “**E**LPG” motif, upstream of the β4 strand for the E60K substitution, the “L**T**IS**G**KRE” motif on the β5 strand for the T79V and G82V substitutions, and the “**R**SERSYGSFR” motif on the β6/β7 strands for the R99Dsubstitution^25^.

The variation of the membrane fluidity of liposomes formed by lipids extracted from *O. oeni* cells was measured by steady-state fluorescence anisotropy of DPH in the absence or presence of Lo18 or modified protein following: (i) temperature ramping (15 °C to 63 °C, with steps of 2 °C/min), (Fig. 1B–F), and (ii) heat shock at 45 °C (Fig. 1G–K).

Concerning the thermal ramping, all the conditions (with or without sHSP) were similar at 15 °C, with an anisotropy value of around 0.2. Increasing temperature caused the liposomes to fluidize, resulting in a decrease in fluorescence anisotropy ranging from 0.195 to 0.075 for liposomes alone (62% decrease) versus 0.204 to 0.156 (24% decrease) for liposomes in the presence of Lo18 protein. The modified proteins presented significantly different profiles, with lipochaperon activity reduced by 40% and 50% for the T79V and G82V proteins (fluorescence anisotropy ranging from 0.189 to 0.114 and 0.205 to 0.101, respectively), and abolished for E60K with a reduction of around 52% (fluorescence anisotropy ranging from 0.195 to 0.094) similar to conditions without Lo18 or in the presence of lysozyme (negative control). For the R99D protein (modification of the β6/β7 loop), a decrease in fluorescence anisotropy of around 40% was observed (ranging from 0.144 to 0.097) between the beginning and the end of the experiment. However, it should be noted that the starting anisotropy was low and the slope of the curve similar to the slope of the curve obtained with the Lo18 protein (Supplementary data S1). Together these two points suggest a transient modification in lipochaperon activity, particularly at low temperatures.

For the measurement of fluorescence anisotropy following heat shock at 45 °C, three phases can be defined: (i) the equilibration phase, in which the reaction mix consists of liposomes and DPH probe in buffer; (ii) the addition of modified or unmodified sHsp or lysozyme; and (iii) the application of heat shock. Fluorescence anisotropy at the start of the experiment was similar under all conditions (value around 0.15). The addition of Lo18, T79V and G82V proteins significantly increased fluorescence anisotropy by 45%, 30% and 26%, respectively (anisotropy values of 0.149 to 0.205 for Lo18, 0.147 to 0.191 for T79V and 0.161 to 0.203 for G82V), suggesting that the presence of the protein immediately rigidified the liposomes. No significant differences were observed when E60K, R99D and lysozyme were added. Heat shock at 45 °C rapidly fluidized the liposomes, which showed fluorescence anisotropy ranging from 0.205 to 0.159 (22% decrease) in the presence of Lo18, or from 0.155 to 0.091 in the absence of protein (41% decrease). Only the R99D protein showed anisotropy values and a thinning profile similar to those obtained for Lo18 (ranging from 0.178 to 0.163). The addition of E60K, T79V and G82V proteins did not limit fluidization. In the presence of these proteins, the final fluorescence anisotropy fluctuated between 0.128 and 0.124. These values were significantly close to those obtained in the presence of lysozyme (negative control) (Fig. 1, Supplementary data S1).

In brief, following induced amino-acid modifications, lipochaperon activity was either abolished (E60K), decreased (T79V and G82V), or transiently decreased (R99D).

### Key amino-acids for Lo18 chaperone activity

**Figure 2:**
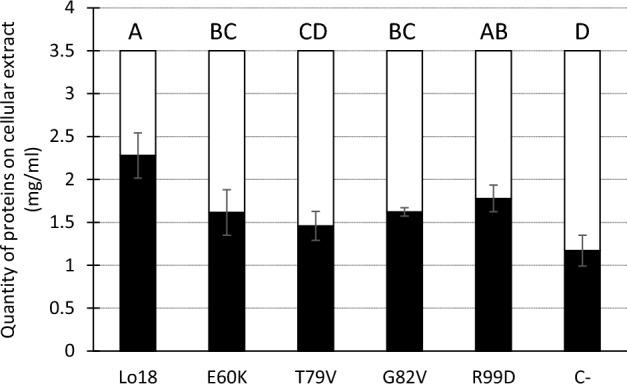
Thermostabilization of proteins from *E. coli* cellular lysates were surproduction of Lo18 or modified protein are induced. A *E. coli* construction with empty plasmid has been used as negative controle (C−). The protein concentration of the lysate was fixe at 3.5 mg/mL, then heat 30 min at 55 °C. The amount of protein unaggregated were measured. The data represent the means and SE of three independent experiments.

The thermostability at 55 °C of a pool of proteins present in an *E. coli* cell lysate was assessed after the overproduction of Lo18 or the modified proteins. The relative quantity of each protein was assessed before and after the induction of heat stress, allowing the comparison of the quantities of protected proteins, which depended on the concentration of the chaperone protein present in the corresponding lysate (Supplemental data S3). In the presence of the Lo18 protein, 35% of the cells in the cell lysate aggregated under heat stress (from 3.5 mg/mL to 2.3 mg/mL). Conversely, lysate protein aggregation reached 67% in the negative control condition (3.5 mg/mL to 1.2 mg/mL). All the modified proteins had a significantly reduced protein aggregation rate compared with the Lo18 condition with the exception of R99D, 53.9% (1.6 mg/mL), 58.3 (1.5 mg/mL), 53.7 (1.6 mg/mL) and 49.2 (1.8 mg/mL) for E60K, T79V, G82V and R99D, respectively. Nevertheless, with the exception of T79V, the protein aggregation rate was significantly higher than in the control condition (Fig. 2). The low rate of protein aggregation during heat stress reflects the chaperone activity of Lo18. As a consequence, apart from R99D, the other modified proteins had reduced (E60K and G82V) or abolished (T79V) chaperone activity.

### Role of these conserved key amino-acids in the structure of Lo18

**Figure 3:**
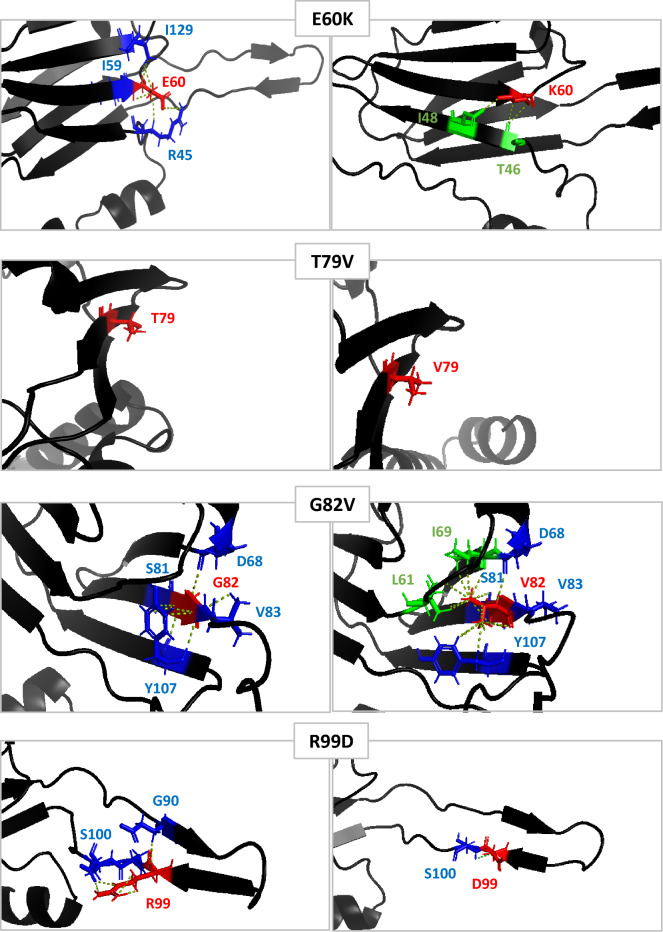
Protein structure modifications induced by point mutation by in silico measurement. Impact of E60K, T79V, G82V and R99D amino acid substitution on predicted 3D Lo18 structure. Substituted amino acids are represented in red, neighbor amino acids able to interact with the initial residues in blue and neighbor amino acids able to interact with substituted residues in green. Interactions between amino acids are represented by yellow dotted line.

In order to explain the change in the lipochaperon activity of the mutated proteins, their different structural levels were analyzed. In silico measurements of the distances between the modified amino-acids (E60K, T79V, G82V and R99D) and their adjacent amino-acids showed a modification of these distances, affecting the probability of their putative interactions. One exception was the T79V substitution for which no major modification was predicted (Fig. 3). For the E60K substitution, the distance of the glutamic acid residue at position 60 (E60-red) allowed binding with three amino-acids (blue) present on the β2 and β9 strands (Fig. 3). Once modified with lysine (60 K), these interactions lapsed and were replaced by a possible interaction with 2 other amino-acids on the β9 strand (green) (Fig. 3). The impact of the G82V substitution on the distance interaction of adjacent amino-acids was very pronounced. Glycine at position 82 (G82-red) could possibly bind to four amino-acids (blue) present on strands β4, β5, and β7. The substituted valine (82 V) created two potential additional interaction sites with two amino-acids present between the β3 and β4 strands and on the β4 strand (green). However, even if the distances between amino-acids were consistent with an interaction, not all of these amino-acids could bind to the residue at position 82 due to the availability of binding sites. In addition, the presence of valine created a stearic imbalance that resulted in the rotation of the aromatic rings of tyrosine at position 107 (Y107) (Fig. 3). Because this tyrosine has been described as linking the two planes of the α-crystallin domain together^25^, the G82V substitution could disrupt the β -sandwich structure of the α-crystallin domain. In the R99D substitution, the aspartic acid in position 99 (99D-red) was no longer accessible to interact with the aspartic acid in position 90 (D90-blue) present on the β6 loop, compared to the original arginine (R99-red) (Fig. 3). At least the distances between E60, G82 and Y107 residues was correct, to allow their interaction with each other, in order to stabilize the monomer structure.

**Figure 4:**
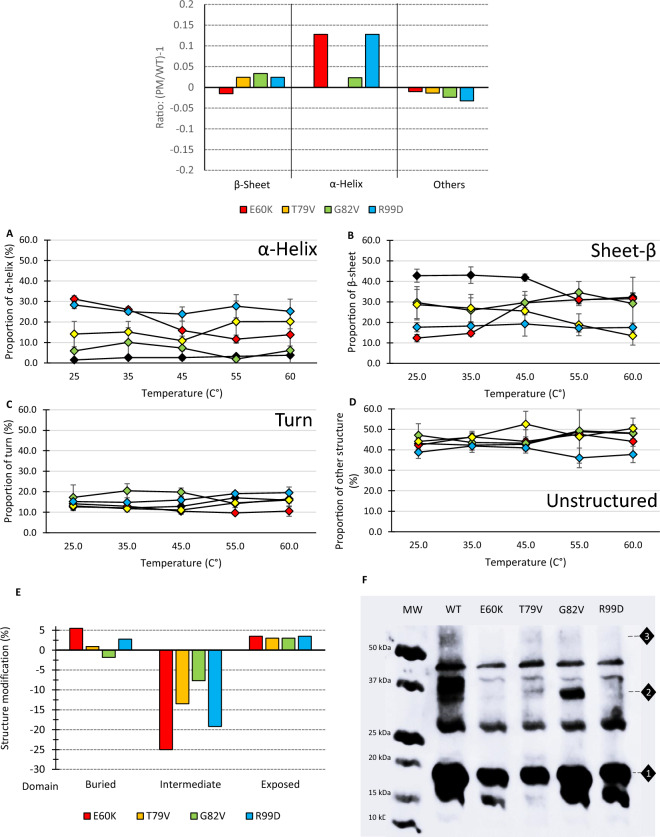
Measuring the impact of point modifications on the structure of proteins. In silico prediction of secondary structure of the modified protein by PredictPortein (**A**)Structure modification of Lo18 (black), E60K (red), T79V (yellow), G82V (green) and R99D (blue) induced by thermal ramping (25 °C to 50 °C on the four types of secondary structures, (**B**) α-helix, (**C**) β-sheet, (**D**) turns and (**E**) other secondary structures, evaluated by SRCD. The data represent the means and SE of three independent experiments. (**F**) Prediction of the impact of point mutation substitution on secondary structure accessibility. (**G**) Oligomeric structure of Lo18 WT or modified. Immunolabelling of Lo18 after in vivo cross-linking. The diamond-shaped numbers from 1 to 3 indicate the presence of monomer, dimer and trimer respectively.

To describe further the changes in the secondary structure of these proteins, in silico prediction and synchrotron radiation circular dichroism (SRCD) experiments were conducted on the Lo18 and modified proteins during temperature elevation (Fig. 4).

The structural predictions made on the modified proteins suggested that all the point mutations have an impact on the secondary structure of the Lo18 protein, with marked structural modifications for the modified E60K and R99D proteins. Indeed, an increase in the number of α-helix around 12% was predicted for these two proteins (Fig. 4A).

SRCD measurement backed up the in silico observation obtained. Each point mutation affected the structure of the protein slightly (for G82V) or strongly (for E60K and R99D) (Fig. 4B–E). The secondary structures of Lo18 and G82V are relatively close. On the contrary, the T79V protein shows few modifications compared with Lo18, while the E60K and R99D proteins are very strongly impacted (Table 1). More precisely, the α-helices and β-sheets were the main structures impacted by these substitutions. A moderate increase in α-helices for T79V (14.1% at 25 °C and 20.2% at 60°) and a significant increase for proteins E60K and R99D (31.2 and 28.4% at 25 °C and 13.8 and 25.2% at 60 °C respectively) was observed. Conversely, up to 45 °C, the proportion of β-sheet was significantly higher for Lo18 (around 42%) than for the modified proteins (18.8% for E60K, 28.4% for G82V, 25.7% for T79V and 18.3% for R99D). From 55 °C onwards, the proportion of Lo18 β-sheet decreased to be similar to the modified proteins at 60 °C. The turns and the non-ordered protein structures (other structures) were less impacted for the point mutations with (Fig. 4; Table 1).

**Table 1:** SRCD measurement of secondary structure (α-helix, β-sheet, turn and other structures) of Lo18 or modified proteins.

a-Helix (%)/ Temperature (°C)	25	35	45	55	60
Lo18	1.5 ± 0.4^A^	2.6 ± 0.2^A^	2.7 ± 0.5^A^	3.2 ± 0.2^A^	3.8 ± 0.9^A^
E60K	31.2 ± 1.6^C^	26.1 ± 1.2^C^	15.9 ± 7.8^BC^	11.7 ± 2.0^B^	13.8 ± 5.5^ABC^
G82V	5.9 ± 7.3^AB^	10.1 ± 7.1^AB^	7.3 ± 5.8^AB^	1.9 ± 1.7^A^	6.1 ± 10.6^AB^
T79V	14.1 ± 6.2^B^	15.1 ± 5.2^B^	10.9 ± 4.9^B^	20.1 ± 10.2^BC^	20.2 ± 5.5^BC^
R99D	28.4 ± 1.8^C^	25.1 ± 1.3^C^	23.9 ± 3.5^C^	27.7 ± 5.6^C^	25.2 ± 6.0^C^

3-sheet (%)/Temperature (°C)	25	35	45	55	60
Lo18	42.8 ± 3.2^A^	43.1 ± 4.0^A^	41.9 ± 1.7^A^	30.9 ± 1.9^A^	32.3 ± 2.2^A^
E60K	12.3 ± 1.8^B^	14.6 ± 1.3^B^	29.5 ± 3.7^B^	31.1 ± 2.9^A^	31.5 ± 2.5^A^
G82V	29.7 ± 7.8^C^	25.9 ± 6.1^C^	29.7 ± 5.4^B^	34.6 ± 5.3^A^	29.2 ± 12.8^A^
T79V	28.6 ± 7.4^C^	27.0 ± 6.7^C^	25.5 ± 1.8^BC^	18.9 ± 5.3^B^	13.5 ± 4.6^B^
R99D	17.6 ± 2.2^D^	18.2 ± 0.8^D^	19.2 ± 5.9^C^	17.2 ± 1.9^B^	17.5 ± 2.2^AB^

Turn (%)/ Temperature (°C)	25	35	45	55	60
Lo18	12.6 ± 1.3^A^	12.1 ± 1.9^A^	12.8 ± 1.2^A^	17.1 ± 0.7^A^	15.9 ± 1.4^A^
E60K	14.1 ± 1.1^A^	12.9 ± 1.3^AB^	10.5 ± 1.4^B^	9.7 ± 0.9^C^	10.6 ± 2.5^A^
G82V	17.3 ± 6.0^A^	20.5 ± 3.4^C^	19.8 ± 2.0^C^	14.2 ± 3.8^B^	16.5 ± 5.8^A^
T79V	13.2 ± 2.3^A^	11.7 ± 0.9^A^	11.1 ± 1.0^B^	14.5 ± 2.2^B^	15.9 ± 3.2^A^
R99D	15.2 ± 1.0^A^	14.8 ± 0.9^BC^	16.0 ± 1.4^C^	19.1 ± 1.1^A^	19.6 ± 0.3^A^

Other structure (%)/Temperature (°C)	25	35	45	55	60
Lo18	43.1 ± 2.1^A^	42.2 ± 2.3^A^	42.7 ± 1.1^A^	48.8 ± 1.5^A^	48.0 ± 0.4^A^
E60K	42.3 ± 2.0^A^	46.3 ± 1.7^A^	44.1 ± 5.7^A^	47.6 ± 2.0^A^	44.1 ± 5.4^A^
G82V	47.1 ± 5.6^A^	43.5 ± 4.8^A^	43.2 ± 2.3^A^	49.3 ± 0.2^A^	48.2 ± 4.0^A^
T79V	44.1 ± 1.7^A^	46.2 ± 2.8^A^	52.5 ± 6.2^A^	46.5 ± 13.0^A^	50.4 ± 5.0^A^
R99D	38.8 ± 3.0^A^	41.9 ± 2.0^A^	40.9 ± 1.4^A^	36.0 ± 4.8^A^	37.8 ± 4.0^A^

Data corresponding to means ± SE and statistical analysis. Measurement were replicat three time and analyse by Kruskal–Wallis statistical test (*P*-value < 0.05).

All these structural changes taken together suggest that the slightest modification could impact the whole structure of the protein and putatively its functionality. Indeed, a prediction of this structural change suggests that the most affected secondary structure proteins (E60K and R99D) are more likely to undergo variation in exposure to solvent (Fig. 4F).

Finally, the quaternary structure of the Lo18 or modified proteins was monitored by immunolabelling using an *E. coli* heterologous system (Fig. 4G). For the Lo18 protein, several bands were observed corresponding to different oligomerization states (oligomer, dimer, trimer) of the protein with complete and/or truncated forms^21,26^. The modified proteins also showed numerous bands corresponding to these multimeric forms. It could be seen that E60K, T79V and R99D were able to produce very low quantities of dimeric forms.

The structural data set demonstrated an effect of the point mutations on the monomeric structure of Lo18 and consequently on its multimeric structure.

### Modification of the Lo18 structure in the presence of membrane lipids

**Figure 5:**
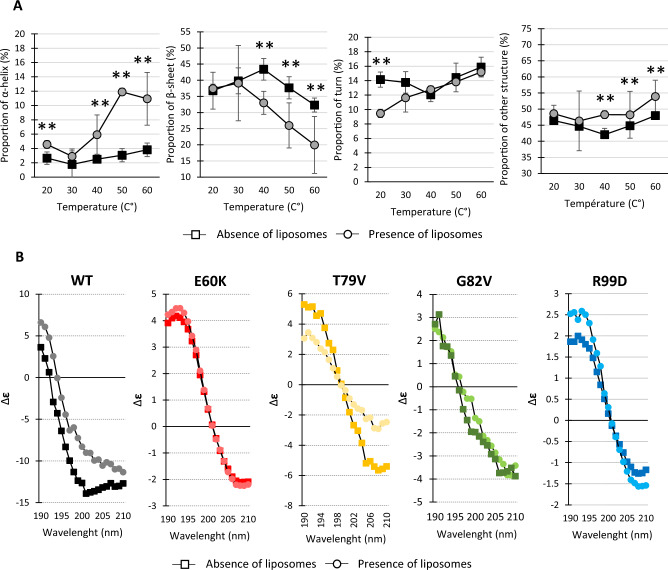
Impact of Lo18/liposomes interaction on protein secondary structure. (**A**) Structure modification of Lo18 WT induce by thermal slope (20 °C to 60 °C) on the four main secondary structures α-helix, β-sheet, turns and other secondary structure in presence (black square) or absence (grey dot) of liposomes. The data represent the means and SE of three independent experiments. (**B**) SRCD spectra on 190–210 nm wavelenght for Lo18 WT (black), E60K (red), T79V (yellow), G82V (green) and R99D (blue) in presence (dark square) or absence (light dot) of liposomes at 25 °C.

To further characterize the changes in the secondary structures of the protein and their potential impact on lipochaperon activity, SRCD experiments were performed during a thermal ramp from 20 °C to 60 °C. For Lo18 in the presence of liposomes, a decrease in the proportion of β-sheets in favor of α-helices, and unordered structures was observed whatever the temperature tested (Fig. 5A). For example, at 45 °C (shock anisotropy temperature), the proportion of β-sheets decreased from 40 to 28% in the presence of liposomes; on the contrary the proportion of α-helices and non-ordered structure increased from 3 to 9% respectively and from 42 to 48% with the addition of liposome (Fig. 5A).

In addition, the analysis of the spectra obtained by SRCD of the Lo18 protein showed that in the presence of liposomes a distinct shoulder appeared in the 195–210 nm range, with a signal elevation of about 35% at 201 nm (lowest point of the spectrum) at 25 °C compared to the condition without liposomes (Fig. 5B). This phenomenon could indicate the insertion of the protein into the lipid bilayer of the liposome.

Furthermore, the interaction between the liposomes and the modified proteins was monitored and compared to Lo18. At 25 °C, no distinct shouldered spectra in the presence of liposomes were observed for E60K and R99D (Fig. 5B). Finally, when insertion occurred, the presence of liposomes stabilized the protein structure upon temperature elevation (Supplemental data S2).

All together, these results highlight the modification of the secondary and tertiary structures of the Lo18 protein after interaction with a lipid bilayer by putatively exposing specific Lo18 domains able to interact with the membrane.

## Discussion

Stress proteins such as small heat shock proteins (sHSP) are present in almost all organisms. Although they have all been described for their role as molecular chaperones^7,27–29^, a growing number of studies have shown that some of them also play the role of lipochaperon by acting at the cell membrane level to maintain its integrity^16,20–24, 30–32^. Few sHSPs have been described as performing both these activities, the mechanisms involved in lipochaperon activity are still very poorly understood. A recent study has shown that certain residues are highly conserved in sHSPs with lipochaperon activity^25^. In this context, the interaction between Lo18 sHsp and *O. oeni* membrane lipids was studied on the basis of the structure–function relationship (Table 2). 2).

**Table 2:** Synthesis table of chaperon and lipochaperon activities of Lo18 or modified proteins link to their secondary structure modifications. The presence or absence of Lo18 serves as a reference for these activities, where “++” indicates a high level, “+” denotes a moderate level, and “–” signifies no activity.

	Lo18	E60K	T79V	G82V	R99D	Ø Lo18
Chaperon activity	++	+	−	+	++	−
Lipochaperon activity slope	++	−	+	+	++	−
Lipochaperon activity shock	++	−	−	+	++	−
Membrane rigidification by protein addition	++	−	++	++	−	−
Membrane insertion (SRCD)	++	−	+	+	−	−
α-Helix	−	++	+	−	++	
β-Sheet	++	−	+	+	−	
Turn	=	=	=	=	=	
Other structure	=	=	=	=	=	

The impact of four of these highly conserved residues (E60, T79, G82 and R99) was assessed for both Lo18 chaperone and lipochaperon activities. With the exception of the modified protein R99D, the chaperon and lipochaperon activities of the modified proteins were reduced (T79V and G82V) or abolished (E60K). Interestingly, the loss of lipochaperon activity of the modified proteins did not correlate with the loss of chaperone activity, as previously demonstrated for the Y107A (loss of lipochaperon activity only) and A123S (loss of chaperone activity only) proteins^24^. The E60K protein, which no longer had a lipochaperon activity, still possessed a chaperone activity, and vice versa for the T79V protein. These results support the hypothesis that key residues are required for the activity of sHSPs, either chaperone or lipochaperon. Nevertheless, the importance of conserved residues in Lo18 activities appears to be strongly correlated with their location on the protein. Indeed, only modifications of residues present on the core (E60K, T79V, G82V) generated a loss of activity, as was already the case for A123S and Y107A proteins^24^. In contrast, modifications of residues present on the long β5/β6 loop, did not cause the loss of chaperone activity (E81A and R82G of HspH from *Bradyrhizobium japonicum*^9^ and R99D from *O. oeni*) and lipochaperon activity (R99D). The modification of the protein's structure could explain these losses of activity, as has often been described for chaperone activity on sHSPs. For example, the structural modification of the α-crystallin domain has an impact on chaperone activity^33,34^ as has been described for human HSPB1^35^, HSPH from *B. japonicum*^9^ and plant sHSPs Ta16.9 and Ps18.1, from *Triticum aestivum* and *Pisum sativum* respectively^36^. While the link between the structure of sHSPs and their lipochaperon activity has yet to be fully demonstrated, it is highly likely that structural modifications also affect their lipochaperon activity.

The secondary to quaternary structures of the various modified proteins were studied to further investigate the structure–function relationship. Predicted structural changes occurring in silico were confirmed in vitro by SRCD experiments for all the modified proteins tested. In general, a decrease in the proportion of β-sheets in favor of an increase in α-helices was observed, suggesting that the amino-acids of interest present in the core protein domain help to maintain the secondary structure of both planes of the Lo18 protein. These modifications are more or less marked with a very strong alteration of the E60K protein and much less significant modifications for the G82V protein. With regard to residue R99, the significant increase in the proportion of α-helices could reflect a relaxation of the β-sheets present on the β6/β7 loop^37^. Indeed, the change in charge induced by the replacement of arginine ® by aspartic acid (D) locally modifies electrostatic interactions, particularly with aspartic acid at position 90. The decrease in these interaction forces will affect the formation of hydrogen bonds between the β6 and β7 sheets of the loop, enhancing their folding into α-helices^38,39^. Similar results have been described following point mutations in the long loop of the human HSPB1 protein (E130K point mutation)^40^. Likewise, point mutations in the core of the human γD-crystallin protein, whose structure and function are close to α-crystallin (to which sHSPs belong), have also been observed^41^. Immunostaining results confirm that modifications of the secondary structure led to an alteration of the quaternary structure. With the exception of G82V, which is weakly modified at the level of its secondary structure, the dimeric structure formation of the other modified proteins is strongly altered, which may explain changes in activities^23,42^.

To get a better understanding of the possible relation between the structural modification of the studied proteins and their lipochaperon activity, the impact of the presence of lipids was studied in more detail. The addition of liposomes to the Lo18 protein also promoted a decrease in the proportion of β-sheets in favor of α-helices. These results suggest that interaction with liposomes increases the relaxation of the protein, as described for HSP16.3 from *M. tuberculosis*^43^. In the presence of lipid bilayers (liposomes) the Lo18 protein spectra present a decrease of the 208 nm negative absorption and a batho-chromic (red-) shift of the π–π*electronic transition, which most likely reflects protein insertion into the bilayer^44,45^. Indeed, the spectra obtained by SRCD, and the size and concentration of the liposomes, allowed obtaining a signal similar to the classical one obtained in oriented circular dichroism, a state-of-the-art technique for studying protein insertion into a membrane^46^. For the modified proteins, the SRCD spectra obtained in the presence of liposomes were different from those obtained for the Lo18 protein. The modified E60K and R99D proteins were no longer able to enter the bilayer, which was reflected by the retention of their negative absorption at 208 nm, while the T79V and G82V proteins could still be inserted. The anisotropy results for the latter two proteins confirmed this insertion, with rapid rigidification of the liposomes as soon as the proteins were introduced.

In conclusion, understanding the mechanisms involved in the interaction between cell membranes and sHSPs are important not only for fundamental knowledge, but also for their applications, especially in the health sector. For example, the results could be applicable to two human sHSPs (HSPB1 and HSPB5) already studied for their therapeutic role in the brain through the spectrum of their chaperone and lipochaperon activity^47–49^. In this work, the changes of 4 amino-acids induced a modification in lipochaperon activity that could be due to a structural protein rearrangement or a direct effect of amino-acids changes. For the three residues (E60, T79 and G82) present in the core of the protein, the greater the structural modifications the greater the decrease on lipochaperon activity. On the contrary, these amino-acid changes seemed to affect chaperone activity to a lesser extent. In contrast, the amino-acid modification present on the β5/β6 loop induced a major change in the protein's secondary structures but no major impact on chaperone and lipochaperon activities were measured. Thus, it is necessary in the future to elucidate the fine regulatory mechanisms involved.

## Material and methods

### Media and growth conditions

*O. oeni* ATCC BAA-1163 was grown at 28 °C and at pH 5.3 in FT80 medium modified by the addition of meat extract instead of casamino acids^50^. *E. coli* BL21 star (DE3) cells were transformed with the plasmids pET28a-hsp18-HIS, pET-E60K, pET-T79V, pET-G82V and pET-R99D, named respectively *E. coli* Lo18 and *E. coli* E60K, *E. coli* T79V, *E. coli* G82V and *E. coli* R99D, and were grown aerobically at 37 °C in Luria–Bertani (LB) medium broth supplemented with 50 μg/mL kanamycin (Sigma, Saint-Louis, US).

### Strain and plasmid construction

The *hsp18* gene was generated from the plasmid pET-*hsp18* 27 by PCR amplification using two primers: Lo18N-NdeI 5′-GCACAGCATATGGCAAATGAATTAATGGATAGAAATGATGG-3′ (forward) and Lo18C-HindIII 5′-TTGGCTAAGCTTTTATTGGATTTCAATATGATGAGTTTGACTTTCG-3′ (reverse), respectively. The coding region of the *hsp18* gene was then cloned into the NdeI and HindIII sites of the expression vector pET28a (Invitrogen, US). The resulting plasmid, designated pET-hsp18-HIS, contains the unmodified complete *hsp18* coding region with a sequence coding the hexahistidine tag in 5’, under the control of the inducible promoter T7.

Site-directed mutations leading to single amino-acid exchanges in Lo18 were introduced by primer-based mutagenesis, using pET-hsp18-HIS as a template. The *hsp18* gene was modified by PCR using specific primers E60K, T79V, G82V and R99D containing a point-nucleotide mutation (Supplementary data, Table S4). The template plasmid was eliminated using a *Dpn1* restriction enzyme (BioLabs) at 10U/µL. Chemically competent *E. coli* BL21 Star (DE3) cells were transformed with the resulting vectors (pET-E60K, pET-T79V, pET-G82V and pET-R99D), according to the manufacturer’s instructions (Invitrogen, US).

### Protein expression and purification

For all the strains (overexpressing proteins Lo18, E60K, T79V, G82V and R99D), cell-free extracts were prepared from 1 L culture of *E. coli* cells grown at 37 °C in LB medium supplemented with kanamycin (50 mg/mL). The production of Lo18 or modified protein was induced by adding 50 μM IPTG (isopropyl β-D-thiogalactopyranoside) for 3 h at 37 °C and shaking. The bacterial solution was centrifuged for 10 min at 4000 g to harvest the cells. The cells were then washed twice and resuspended in 60 mL of buffer A (0.2 mM phosphate buffer pH 7.0, 500 mM NaCl). The cells were then disrupted over 3 cycles at 8 kPSI using the Continuous Flow Cell Disrupter (Constant Systems Ltd, UK). The resulting cell lysate was centrifuged for 1 h at 4 °C at 12,000 *g* to remove the cellular debris present.

Cell lysates containing Lo18 or modified protein were purified by affinity chromatography using a HisTrap HP column, 1 mL (Cytiva, US). To do this, 5 mL of cell lysate were injected into the column previously equilibrated with 10 mL of buffer A with a flow rate of 1 mL/min. An elution gradient was set up with 20 mL at a flow rate of 1 mL/min to reach a 100% concentration of buffer B (0.2 mM phosphate buffer, 500 mM NaCl, 100 mM imidazole, pH 8). The 100% buffer B concentration was maintained for 10 mL with a flow rate of 1 mL/min. The purified protein was then stored at -20 °C. An additional dialysis step was added before the circular dichroism experiments. Thus, the samples were dialyzed against 30 times the volume of sodium phosphate buffer pH 7.0, using an Ultracel amicon with a 10 kDa cut-off (Merck, US).

### Assay for Thermostability of Lo18 or modified protein

Cellular lysate for all the strains (overexpressing proteins Lo18, E60K, T79V, G82V and R99D) were produced as described above. In order to test the thermostability of *E. coli* cellular proteins, the total protein concentration of the lysates was measured by the Bradford assay (Biorad, France) and adjusted to 3.5 mg/mL on phosphate buffer (pH 7). To measure protein denaturation, lysates were heated at 55 °C for 30 min and centrifuged at 12 000 g for 10 min. The amount of protein in the supernatant fractions (corresponding to unaggregated proteins) was measured by the Bradford assay.

### Structure analysis

#### In silico structure prediction and protein membrane interaction

The secondary structure of Lo18 and modified proteins was predicted using the PredictProtein online software. Then, models of the tertiary structure of these proteins were generated using trRosetta and Alphafold2 online software. The impact of amino-acid present on the point mutation of all the models obtained was analyzed using the measurement functionality of PyMOL 4.60 software.

Molecular Dynamic simulations (MD) in explicit water were performed using initial structures: A0NLC2 truncated of the first 25 amino-acids of the N-terminal domain^51^, and total structure obtained using trRosetta and Alphafold2 online software. All unbiased simulations were carried out with the GROMACS software package^52^. Na^+^,Cl^–^ ions were added at a concentration of 0.1 M. The initial velocities were chosen randomly. The system was warmed up for 40 ps and equilibrated for 600 ps with lower restraints, finishing with no restraints at 310 K. We performed different MD runs which simulated between 1 and 2 μs the interactions of the proteins to 360 K, 385 K, 410 K, 435 and 460 K. These simulated temperatures corresponded to the behaviors (unfolding) of the Lo18 dimer observed at the temperatures between 12 °C and 87 °C.

#### Synchrotron radiation circular dichroism spectroscopy

Circular dichroism spectra were collected on the DISCO beamline (Synchrotron SOLEIL, France). In detail, the instrument was calibrated using a 99% pure (+) camphor-10-sulphonic acid (Sigma Aldrich, Saint-Louis, US) at 25 °C after each beam fill^53^. Protein samples (Lo18 or modified proteins) were prepared in 50 mM sodium phosphate buffer, pH 7.0. Then, 50 µL of the sample was loaded into a 0.02 cm pathlength demountable cylindrical quartz Suprasil cell (Hellma, Germany) and subjected to a thermal scan from 15 °C to 84 °C with a step of 3 °C. Each dataset was collected in 1 nm steps from 262 to 176 nm, with an integration time of 1.2 s and a spectral bandwidth of 1 nm. Three scans of each sample and the equivalent baseline were collected. At each temperature step the three collected spectra were averaged and smoothed (9 points with Savitzky-Golay smoothing), and the baseline subtracted with the averaged and smoothed spectra of the buffer without protein taken at 15 °C. Spectra were then zeroed between 250 and 260 nm. All spectra were normalized according to the protein concentration and pathlength using the CDToolX software.

A similar protocol was used in the presence of liposomes (preparation described below) using 250 µM liposomes and 140 µM proteins, with a thermal scan from 25 to 55 °C with a step of 5 °C. Deconvolution of the spectra was performed with the BeStSel server^54^.

### Cross-linking

The oligomeric statuses of Lo18 and modified protein were analyzed by cross-linking experiments in vivo. Measurements were performed as described by Delmas et al.^26^. Therefore, cellular extracts were treated with formaldehyde, to obtain a final concentration of 1% (w/w). Five µg of cross-linked samples were separated onto 12% SDS-PAGE gel before Western blot analysis using antibodies against Lo18.

### Lipid interaction

#### Preparation of liposomes

Lipids from an *O. oeni* culture in exponential phase were extracted and purified according to Bligh and Dyer and Maitre et al.^22,55^ with slight modification. A film of extracted lipids was obtained by evaporating chloroform using a nitrogen flux. Then, a pre-warmed 50 mM phosphate buffer pH 7.0 at 55 °C was added to the sample and gently mixed. The lipid solution was sonicated twice for 2 min (Branson Ultrasonics™ CPX-952-138R, Branson Ultrasonics, Brookfield, CT, US) and rehydrated for 4 h at 55 °C. The lipid particles were then extruded through a polycarbonate membrane with 1 μm diameter pores to obtain the liposomes which were stored at 4 °C for a maximum of 1 week.

#### Fluidity measurements

Membrane fluidity was monitored by fluorescence anisotropy measurement using a Fluorolog 3 spectrofluorimeter (FLUOROLOG-3, Jobin Yvon Inc, USA). The anisotropy values (inversely proportional to the membrane fluidity) were calculated according to Shinitzky and Barenholz^56^. Excitation and emission wavelengths were 360 nm and 431 nm, respectively. The measurement was performed for 30 min (1 determination every 10 s) in a quartz cuvette filled with 250 μL of liposomes prepared as described above, with 3 μM of 1,6-diphenyl-1,3,5hexatriene (DPH) as a probe (Sigma Aldrich, Saint-Louis, US). Data were recorded in the presence or absence of Lo18 or modified protein, using a mass ratio of 1:2 (m/m) between the sHSP and the liposomes. After inserting the probe (10 min at 10 C), a linear temperature gradient from 15 to 63 °C (increase of 2 °C per min) controlled by a Peltier system (QNW TC1 temperature controller, Quantum Northwest, Liberty Lake, WA, USA) and heat shock at 45 °C for 15 min were applied to the liposome suspension. Each experiment was done in triplicate.

### Statistical analysis

At least three independent measurements were performed for each of the conditions tested on fluorescence anisotropy, thermostability and SRCD measurement. Statistical analyses were performed by the statistical RStudio software (version 1.2.5033). The normality of the distribution and homogeneity of the variances of each condition were tested by the Shapiro–Wilk test and the Bartlett test, respectively. Then, a non-parametric Kruskal–Wallis test was used to compare the samples with a significance level of α = 0.05. All the statistical tests were considered significant at a *p*-value < 0.05.

### Supplementary Information

Supplementary Information.

## Data Availability

The datasets generated during and/or analyzed during the current study are available from the corresponding author on reasonable request.
