# Supplementary material for: Significant influence of four highly conserved amino-acids in lipochaperon-active sHsps on the structure and functions of the Lo18 protein
Source: Sci Rep. 2023 Nov 3;13:19036. doi: 10.1038/s41598-023-46306-6 (PMC10624808; doi:10.1038/s41598-023-46306-6)
Supplement: Supplementary file 1 — Supplementary Information. [file 41598_2023_46306_MOESM1_ESM.pdf]

		Lo18	Ø Lo18	E60K	T79V	G82V	R99D	Lysosyme
Slope Heat shock	Interaction Lo18/liposomes	0.0139 ± 0.007 DE	-0.0012 ± 0.001 A	0.0031 ± 0.004 CD	0.011 ± 0.002 A	0.0105 ± 0.004 AB	0.0048 ± 0.003 BC	-0.0016 ± 0.001 E
	heat shock	-0.0045 ± 0.001 AB	-0.0045 ± 0.001 C	-0.0035 ± 0.0003 B	-0.0045 ± 0.0004 C	-0.0047 ± 0.0004 CD	-0.0011 ± 0.004 A	-0.0058 ± 0.001 D
Anisotropy values Heat shock	Before the induction	0.141 ± 0.02 AB	0.155 ± 0.006 AB	0.157 ± 0.0005 AB	0.146 ± 0.001 B	0.161 ± 0.005 AC	0.159 ± 0.003 AC	0.181 ± 0.007 C
	Add of Lo18	0.149 ± 0.01 AB	0.16 ± 0.003 A	0.158 ± 0.002 AB	0.147 ± 0.001 B	0.161 ± 0.003 A	0.159 ± 0.004 AB	0.2 ± 0.005 C
	heat shock	0.205 ± 0.008 C	0.155 ± 0.005 A	0.17 ± 0.01 AB	0.191 ± 0.004 BC	0.203 ± 0.007 C	0.178 ± 0.002 AB	0.194 ± 0.006 C
	4 minutes after shock	0.202 ± 0.001 C	0.162 ± 0.003 A	0.18 ± 0.004 AB	0.194 ± 0.002 BC	0.203 ± 0.008 C	0.178 ± 0.009 AB	0.212 ± 0.012 C
	8 minutes after shock	0.155 ± 0.006 B	0.118 ± 0.01 A	0.124 ± 0.001 A	0.123 ± 0.005 A	0.162 ± 0.022 B	0.149 ± 0.012 B	0.143 ± 0.004 B
	12 minutes after shock	0.157 ± 0.004 C	0.095 ± 0.013 A	0.117 ± 0.004 A	0.119 ± 0.003 A	0.129 ± 0.004 B	0.155 ± 0.015 BC	0.133 ± 0.003 BC
	16 minutes after shock	0.16 ± 0.003 D	0.101 ± 0.007 A	0.121 ± 0.005 AB	0.117 ± 0.001 AB	0.131 ± 0.005 BC	0.156 ± 0.017 CD	0.137 ± 0.011 BC
	20 minutes after shock	0.159 ± 0.004 C	0.091 ± 0.009 A	0.124 ± 0.006 B	0.122 ± 0.002 AB	0.128 ± 0.007 B	0.163 ± 0.02 C	0.119 ± 0.008 AB
Anisotropy values Heat slope	15°C	0.204 ± 0.027 A	0.195 ± 0.01 A	0.195 ± 0.009 A	0.189 ± 0.005 A	0.205 ± 0.023 A	0.1443705 ± 0.008 A	0.161 ± 0.014 A
	23°C	0.203 ± 0.009 BC	0.176 ± 0.012 A	0.1678 ± 0.008 BC	0.156 ± 0.004 BCD	0.185 ± 0.007 AB	0.128 ± 0.007 D	0.151 ± 0.011 CD
	31°C	0.204 ± 0.014 B	0.142 ± 0.007 A	0.142 ± 0.005 B	0.163 ± 0.007 A	0.157 ± 0.003 A	0.118 ± 0.006 C	0.132 ± 0.01 BC
	41°C	0.152 ± 0.017 C	0.098 ± 0.012 A	0.117 ± 0.003 BC	0.149 ± 0.004 A	0.126 ± 0.006 AB	0.119 ± 0.007 BC	0.109 ± 0.007 BC
	49°C	0.141 ± 0.018 A	0.104 ± 0.009 A	0.108 ± 0.006 A	0.134 ± 0.003 A	0.119 ± 0.009 A	0.107 ± 0.01 A	0.097 ± 0.011 A
	53°C	0.144 ± 0.016 D	0.079 ± 0.012 A	0.103 ± 0.006 C	0.128 ± 0.002 AB	0.114 ± 0.005 BC	0.104 ± 0.01 CD	0.092 ± 0.009 CD
	61°C	0.152 ± 0.018 B	0.069 ± 0.01 A	0.096 ± 0.008 AB	0.115 ± 0.004 AB	0.104 ± 0.008 AB	0.105 ± 0.008 AB	0.09 ± 0.009 AB
	65°C	0.156 ± 0.031 A	0.075 ± 0.019 A	0.094 ± 0.008 A	0.114 ± 0.005 A	0.101 ± 0.015 A	0.097 ± 0.005 A	0.081 ± 0.009 A

Supplementary data S1 : Measure of fluorescence anisotropy of DPH inserted into *O. oeni* liposomes during thermal ramping between 16 to 64 °C or after heat shock at 45 °C. Data corresponding to means ± SE and statistical analysis. Measurement were replicat three time and analyse by Kruskal-Wallis statistical test (P-value<0.05).

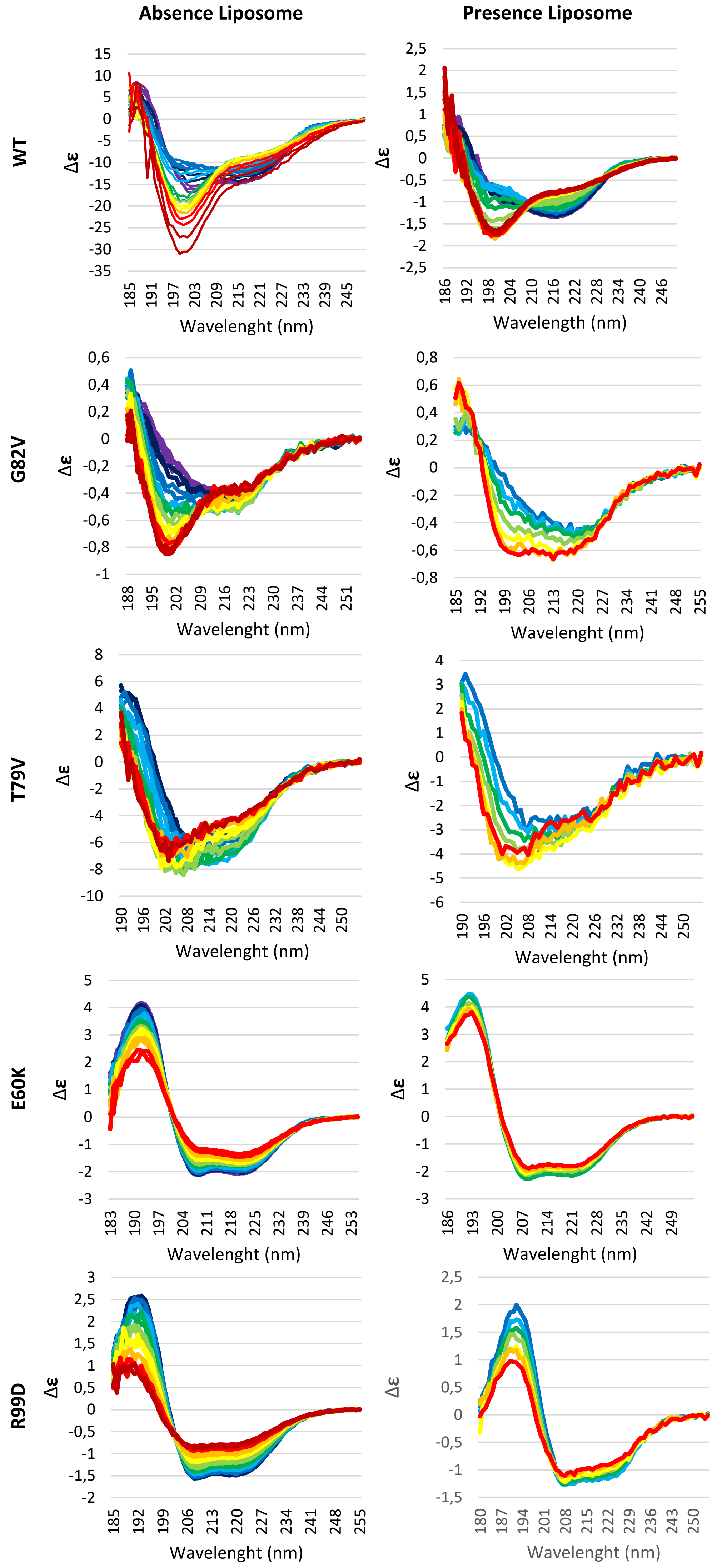

Supplementary data S2: SRCD spectra of Lo18 WT, E60K, T79V, G82V and R99D in presence or absence of liposomes on temperature slope respectively to 25 to 76°C with step of 3°C in absence of liposomes and to 25°C to 55°C with a step of 5°C in presence of liposomes. Color gradient from dark blue to red are correlate respectively from the smallest to the highest temperature of slope.

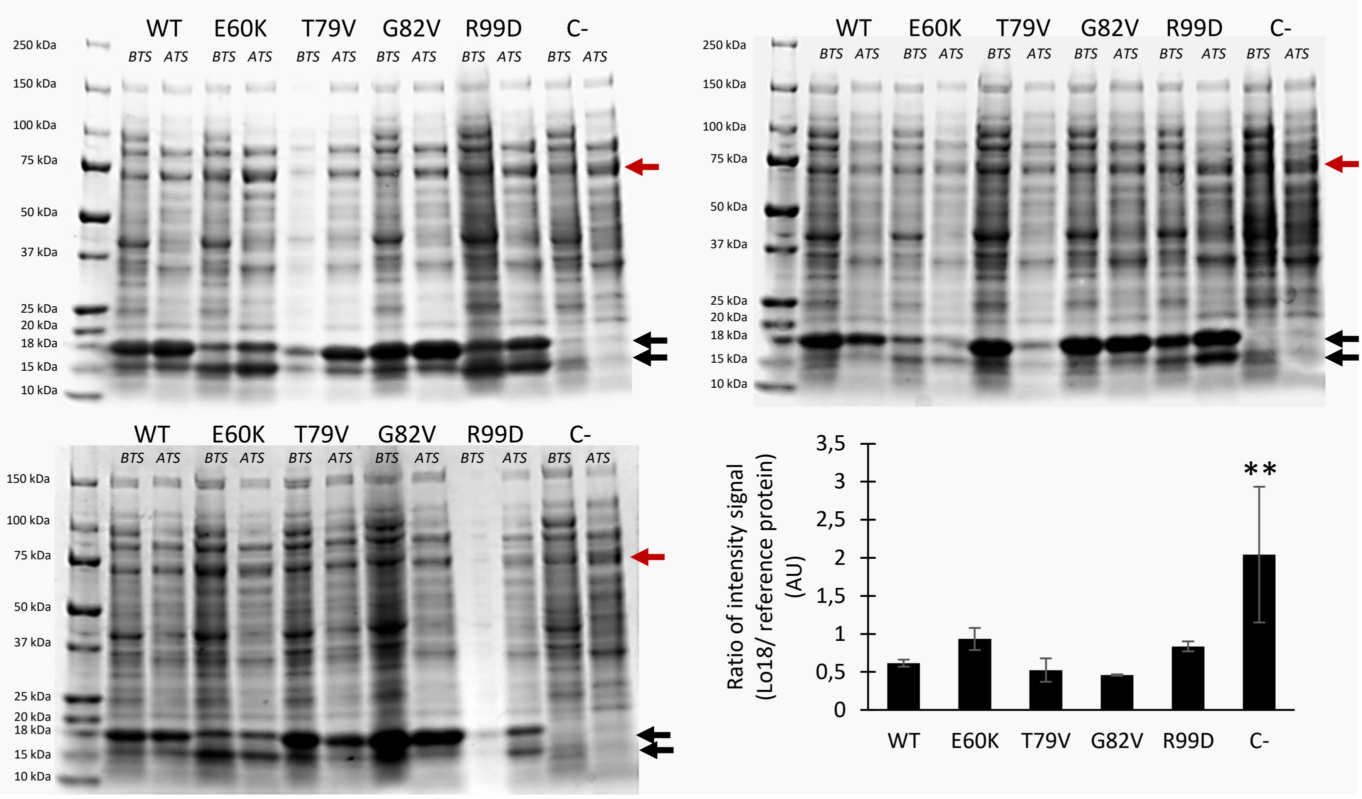

Supplemental data S3: Verification of protein quantity by SDS-page. Each gel represents a replicate of the experiment, and the presence of the protein was checked (respectively in BTS for Before the Thermal Stress and ATS for After the Thermal Stress). The intensity of the bands corresponding to Lo18 (whole or truncated, represented by the two arrows) were normalised with a reference protein (around 75 kDa) in order to avoid variations in the amount of protein between wells, and then analysed statistically (Kruskal-Wallis, nonparametric test, p.value<0.05). Graphic represent means of the ration Lo18/ reference protein +/- SD.

Supplementary data S4: Strains, plasmids and oligonucleotides used in this study.

.

**Strains**	**Relevant characteristic or description**	**Resistance(s)**	**Source or reference**
Escherichia coli			
DH5α	*F*– Φ80*lac*ZΔM15 Δ(*lac*ZYA_*arg*F) U169 *rec*A1 *end*A1 *hsd*R17(r_{k-}, m_k^+) *pho*A *sup*E44 *thi*_1 *gyr*A96 *rel*A1 λ–		[27]
BL21 Star (DE3)	F$^-$*ompT* *hsd*S$_B$ (r_B^-, m_B^-) *galdcmrne*131 (DE3)		[27]
E. coli Lo18	BL21 *hsp18*(OENOO_66120):: pET-hsp18-HIS	Kan^r	This work
E. coli E60K	BL21 *hsp18*(OENOO_66120):: pET-E60K	Kan^r	This work
E.coli T79V	BL21 *hsp18*(OENOO_66120):: pET-T79V	Kan^r	This work
E. coli G82V	BL21 *hsp18*(OENOO_66120):: pET-G82V	Kan^r	This work
E. coli R99D	BL21 *hsp18*(OENOO_66120):: pET-R99D	Kan^r	This work

**Plasmids**	**Relevant characteristic or description**	**Resistance(s)**	**Source or reference**
pET-28a	Insertional vector for lactobacilli	Kan^r	[27]
pET-*hsp18*-HIS	pET-28a with a 447 bp fragment of the gene *hsp18* cloned at NdeI/ HindIII sites	Kan^r	This work
pET-E60K	pET- *hsp18*-HIS point mutated of glutamic acid by lysine at position 60	Kan^r	This work
pET-T79V	pET- *hsp18*-HIS point mutated of threonine by valine at position 79	Kan^r	This work
pET-G82V	pET- *hsp18*-HIS point mutated of glycine by valine at position 82	Kan^r	This work
pET-R99D	pET- *hsp18*-HIS point mutated of arginine by aspartic acid at position 99	Kan^r	This work

**Oligonucleotides**	**Sequence (5'_3')**
Construction of pET-*hsp18*_HIS, pET-E60K, pET-T79V, pET-G82V, pET-R99D	
F_Lo18_His	GCACAGCATATGGCAAATGAATTAATGGATAGAAATGATGG
F_Lo18_E60K	GAATACGGCCTGAAAATCAAACTTCCAGGCTTG
F_Lo18_T79V	CAAATGATAATCTAGTGGTATCAGGAGTTTTGAGTTCCAAGGCC
F_Lo18_G82V	CTAACGGTATCAGTAGTTTTGAGTTCCAAGGC
F_Lo18_R99D	CAAGAAAAATAATGTCGTTGACAGCGAACGTCGC
R_Lo18_His	TTGGCTAAGCTTTTATTGGATTTCAATATGATGAGTTTGACTTTCG
R_Lo18_E60K	CAAGCCTGGAAGTTTGATTTTCAGGCCGTATTC
R_Lo18_T79V	GGCCTTGGAACTCAAAACTCCTGATACCACTAGATTATCATTTG
R_Lo18_G82V	GCCTTGGAACTCAAAACTACTGATACCGTTAG
R_Lo18_R99D	GCGACGTTCGCTGTCAACGACATTATTTTTCTTG

Kan^r, kanamycin resistant.

F_, forward; R_, reverse

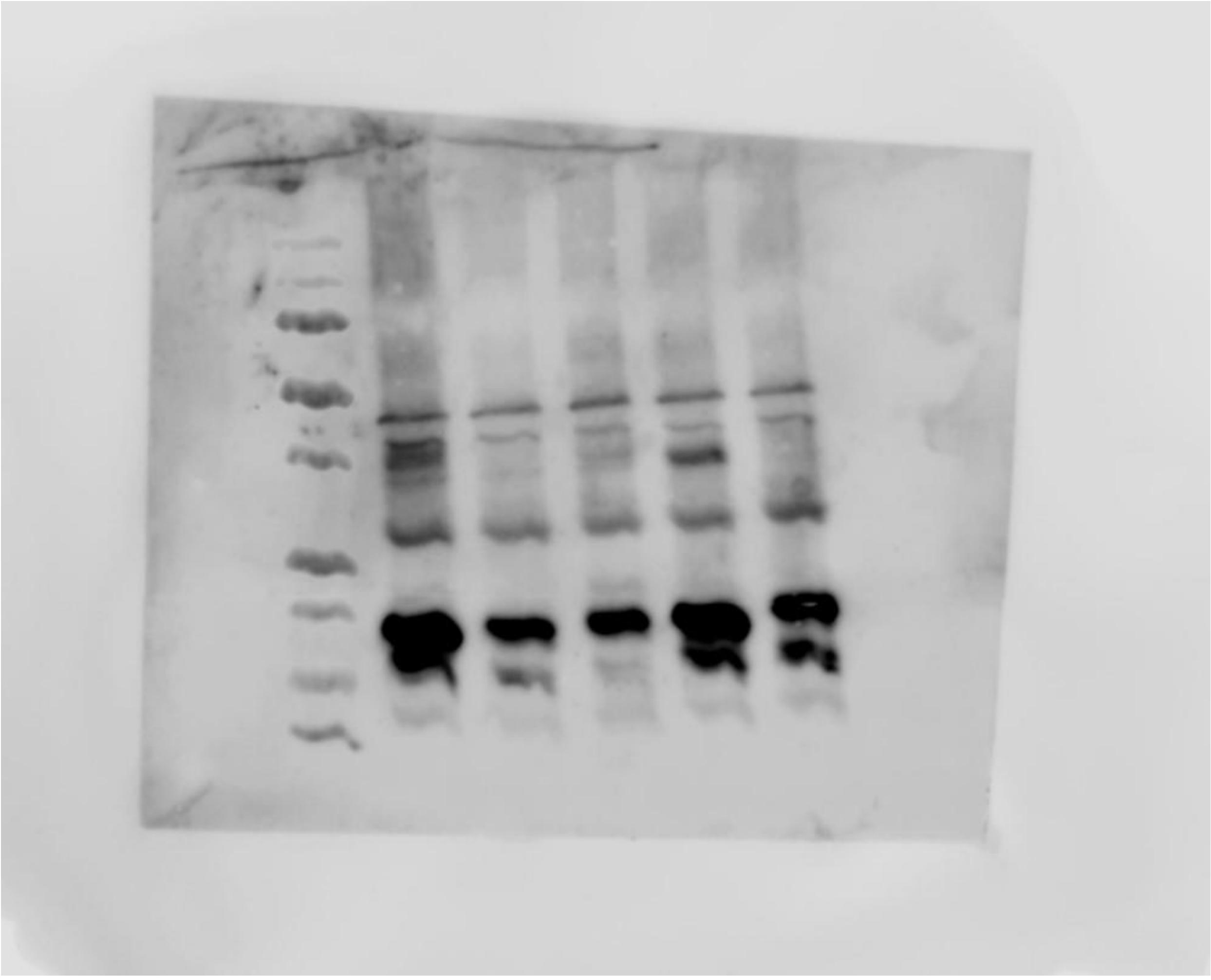

Supplemental data S5: Unprocessed western blot image shown in Figure 4F. The image corresponds to the raw acquisition of the immunolabelling performed in *vivo* on *E. coli* cells used to observe the oligomeric organisation of Lo18.
